# Retraction Note: KIF4A promotes tumor progression of bladder cancer via CXCL5 dependent myeloid-derived suppressor cells recruitment

**DOI:** 10.1038/s41598-026-39297-7

**Published:** 2026-02-10

**Authors:** Ningshu Lin, Luyan Chen, Yunni Zhang, Yi Yang, Lei Zhang, Lei Chen, Peng Zhang, Huiming Su, Min Yin

**Affiliations:** 1 Department of Urology, Xiang’an Hospital of Xiamen University, Xiamen, Fujian People’s Republic of China; 2 Medical Center of Xiamen University, Xiang’an Hospital of Xiamen University, Xiamen, Fujian People’s Republic of China; 3 Department of Urology, Lihuili Hospital, Ningbo Medical Center, Medical College of Ningbo University, Ningbo University School of Medicine, Xingning Road 57, Ningbo, 315040 Zhejiang China; 4 Department of Pathology, Lihuili Hospital, Ningbo Medical Center, Medical College of Ningbo University, Ningbo, Zhejiang People’s Republic of China

Retraction of: *Scientific Reports* 10.1038/s41598-022-10029-x, published online 10 April 2022

The Editors have retracted this Article. After publication, concerns were raised regarding the data presented in Figure 1G and Figure 5O and 5Q. Specifically, two sets of tissue sections in Figure 1G appear to overlap with one another, and portions of the assays in Figure 5O appear to overlap, in some instances after rotations, with portions of the assays in Figure 5Q. The panels in question represent tissues or assays subject to different experimental conditions. The Editors therefore no longer have confidence in the results and conclusions reported in this Article.

The Authors did not reply to correspondence from the Editors about this retraction.

